# Prediction of Bone Marrow Metastases Using Computed Tomography (CT) Radiomics in Patients with Gastric Cancer: Uncovering Invisible Metastases

**DOI:** 10.3390/diagnostics14151689

**Published:** 2024-08-05

**Authors:** Jiwoo Park, Minkyu Jung, Sang Kyum Kim, Young Han Lee

**Affiliations:** 1 Department of Radiology, Research Institute of Radiological Science, and Center for Clinical Imaging Data Science (CCIDS), Yonsei University College of Medicine, Seoul 03722, Republic of Korea; classic0610@yuhs.ac; 2 Division of Medical Oncology, Department of Internal Medicine, Yonsei University College of Medicine, Seoul 03722, Republic of Korea; 3 Department of Pathology, Yonsei University College of Medicine, Seoul 03722, Republic of Korea

**Keywords:** gastric cancer, computed tomography, radiomics, machine learning, bone marrow metastasis, micrometastasis

## Abstract

We investigated whether radiomics of computed tomography (CT) image data enables the differentiation of bone metastases not visible on CT from unaffected bone, using pathologically confirmed bone metastasis as the reference standard, in patients with gastric cancer. In this retrospective study, 96 patients (mean age, 58.4 ± 13.3 years; range, 28–85 years) with pathologically confirmed bone metastasis in iliac bones were included. The dataset was categorized into three feature sets: (1) mean and standard deviation values of attenuation in the region of interest (ROI), (2) radiomic features extracted from the same ROI, and (3) combined features of (1) and (2). Five machine learning models were developed and evaluated using these feature sets, and their predictive performance was assessed. The predictive performance of the best-performing model in the test set (based on the area under the curve [AUC] value) was validated in the external validation group. A Random Forest classifier applied to the combined radiomics and attenuation dataset achieved the highest performance in predicting bone marrow metastasis in patients with gastric cancer (AUC, 0.96), outperforming models using only radiomics or attenuation datasets. Even in the pathology-positive CT-negative group, the model demonstrated the best performance (AUC, 0.93). The model’s performance was validated both internally and with an external validation cohort, consistently demonstrating excellent predictive accuracy. Radiomic features derived from CT images can serve as effective imaging biomarkers for predicting bone marrow metastasis in patients with gastric cancer. These findings indicate promising potential for their clinical utility in diagnosing and predicting bone marrow metastasis through routine evaluation of abdominopelvic CT images during follow-up.

## 1. Introduction

Gastric cancer is a significant contributor to cancer-related mortality worldwide [1]. The incidence of gastric cancer is influenced by the complex interplay between genetics, environment, lifestyle, and dietary habits [2]. Clinically, gastric cancer often presents as a silent and asymptomatic disease in its early stages, contributing to late-stage diagnosis and poor prognosis.

Gastric cancer can metastasize to distant sites, mainly the liver and lungs [3,4]. The skeletal system is also commonly affected. Although some studies report the incidence of bone metastasis in gastric cancer to be approximately only 0.9–2.1% [5], the clinical diagnosis of metastasis may be underdiagnosed, as autopsy findings reveal a higher frequency in the range of 13.4–15.9% [6]. This suggests that bone metastasis may be more common than clinically diagnosed. Furthermore, bone metastases in gastric cancer are associated with poor prognosis [7,8]. The median survival time of patients with gastric cancer and bone metastasis is 3–4 months after the detection of bone metastasis [9]. Consequently, metastatic gastric cancer, particularly in patients with advanced bone marrow metastases, remains a significant therapeutic challenge for medical oncologists because of its association with advanced disease progression [10]. From the perspective of the patient’s quality of life, bone metastasis can cause intractable pain, making early diagnosis and appropriate treatment essential for patients with bone metastases [5]. Therefore, an accurate and timely diagnosis is crucial for guiding treatment decisions and implementing interventions to alleviate symptoms and improve the patient’s overall life quality.

The diagnosis of bone metastasis in gastric cancer typically involves imaging studies such as computed tomography (CT), magnetic resonance imaging (MRI), whole-body bone scans (WBBS), positron emission tomography (PET)-CT, and PET-MRI. Bone-specific markers, such as alkaline phosphatase, may also be elevated in the presence of bone metastases [11]. Among these, abdominopelvic CT (APCT) has been the most commonly used imaging technique since the initial diagnostic workup stage of gastric cancer [12] and can serve as a valuable and widely accessible imaging modality for screening the axial skeleton in these patients [13]. However, CT imaging frequently detects bone metastases in late stages, which are associated with poor outcomes. Furthermore, CT has limitations in identifying small bone metastases in the absence of obvious pathological alterations in the osseous structure; if detected, the imaging findings may be extremely subtle, limiting the certainty of a diagnosis [14]. Owing to these occult initial clinical manifestations, bone metastases can easily be missed or misdiagnosed, potentially increasing the mortality rate [15]. In some advanced cancers, advanced bone metastases reportedly appear before the primary tumor site [16]. The various presentations of bone metastases often fail to reflect the diversity of tumor biology, resulting in a delay in identifying treatment resistance and the chance to make therapeutic modifications [17]. Although advanced imaging methods, including PET-CT and PET-MRI, generally show improvements in diagnostic accuracy for the detection of metastases over conventional CT [18], with the ability to quantify biological processes related to the bone microenvironment and tumor cellularity [19], they are not feasible for continuous regular follow-up examinations from early to late stages in patients with gastric cancer.

For the early diagnosis of bone marrow metastasis prior to gross detection on imaging, clinicians have alternatively performed bone marrow studies on both iliac bones. This has been applied mostly to patients with gastric cancer who have not been previously diagnosed with bone metastasis but present with signs of thrombocytopenia during follow-up, based on the fact that bone marrow infiltration of cancer cells leads to bone marrow structural destruction and hematopoietic dysfunction [20]. However, bone marrow studies are highly invasive compared with imaging techniques and can cause considerable discomfort to the patient. Given that bone metastasis can affect the hematopoietic system before significant changes appear on imaging, this study focused on developing a more sensitive and effective method for diagnosing bone metastasis using APCT, which is the most widely used imaging modality for patients with gastric cancer.

Recent studies have investigated CT radiomics by leveraging texture features [21,22,23]. CT texture analysis provides an objective evaluation by quantifying data that capture information related to lesion attenuation and texture in images, representing tissue heterogeneity, which is often not visible to the naked eye [21]. This has prompted efforts to improve reproducibility, driven by advancements in machine learning and a substantial increase in computational power [22], which have propelled the clinical field of radiomics in recent years [23]. The principle of radiomics involves extracting high-dimensional data from radiological images and analyzing various classes of radiomic features to aid clinical decision making and overcome the constraints of visual interpretation. This approach has the potential to detect pathological findings even in the absence of visible abnormalities [24,25,26,27], maximizing the information obtained from clinical images. By analyzing quantitative information that cannot be visually assessed, radiomics enhances diagnosis and prognostic prediction without the need for further image acquisition [28,29].

To the best of our knowledge, no previous study has evaluated bone metastases and micrometastases using CT-based radiomics in patients with gastric cancer. We hypothesized that the application of CT-based radiomics to bone metastases from gastric cancer may reveal important imaging information that cannot be visually detected by the human eye. Therefore, this study aimed to evaluate the possibility of using CT radiomics as a quantitative imaging biomarker to detect the presence or absence of bone metastasis in patients with gastric cancer.

## 2. Materials and Methods

### 2.1. Patient Selection

This retrospective study was approved by the Institutional Review Board of our hospital, which waived the requirement for informed consent. We searched our picture archiving and communication system-electronic medical record (PACS-EMR) hospital information system to identify patients who met the following inclusion criteria: (1) patients aged ≥ 18 years who had pathologically confirmed gastric cancer at our cancer center from June 2005 to July 2022; (2) patients not diagnosed with bone metastasis by bone biopsy prior to bone marrow studies; (3) patients with thrombocytopenia (cutoff, <150,000/μL) during the follow-up period for gastric cancer who had a pathological diagnostic record of bone metastasis based on blind bone marrow aspiration/biopsy of the iliac bones; and (4) patients with contrast-enhanced APCT image acquisition prior to bone marrow aspiration/biopsy. A total of 173 patients were included in this study. We refined the patient population according to the following exclusion criteria: (1) patients with double primary cancer (*n* = 4; with renal cell carcinoma, hepatocellular carcinoma, small cell lung cancer, and esophageal cancer, respectively); (2) patients whose gastric lesion was not adenocarcinoma (*n* = 25; gastrointestinal stromal tumor, neuroendocrine tumor, or hematologic malignancy); (3) patients pathologically diagnosed with bone metastasis before performing a bone marrow study owing to thrombocytopenia (*n* = 20); (4) patients who did not meet the criteria for thrombocytopenia at the time of bone marrow aspiration (*n* = 25); (5) patient without valid bone marrow aspiration/biopsy results (*n* = 1); (6) patients with only non-contrast CT (*n* = 1); and (7) patients without possible CT segmentation due to severe artifacts (*n* = 1). Finally, 96 patients were included. We collected clinical information, including age at the time of gastric cancer diagnosis, platelet count and date, date of CT scan prior to the bone marrow study, date of the bone marrow study, and corresponding pathologic results.

**Figure 1:**
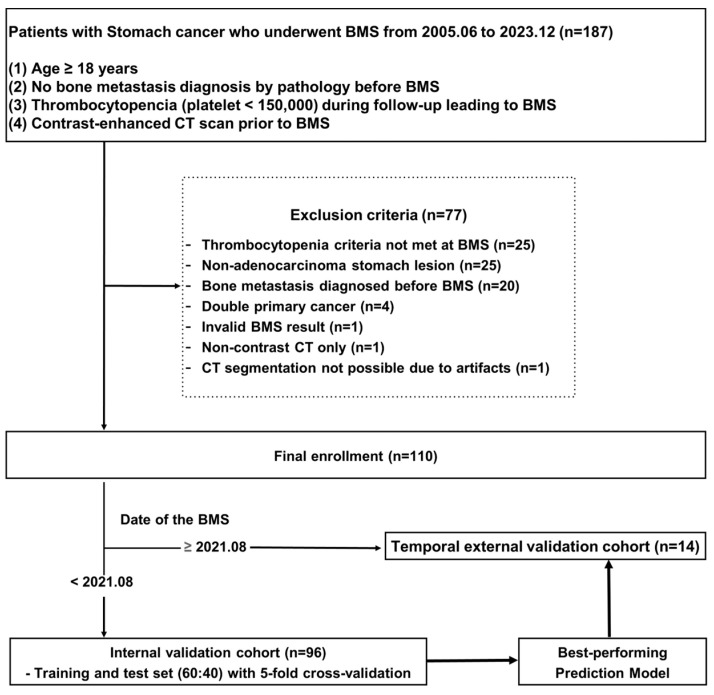
Flowchart for patient selection and development of datasets. Abbreviations: BMS, bone marrow study; CT, computed tomography.

In addition to this study population, this study also included 14 patients who met the inclusion criteria between September 2022 and August 2023 as the temporal external validation group. A summary of the patient selection process is shown in Figure 1.

### 2.2. Tissue Sampling and Pathologic Confirmation

The indication for a bone marrow study was thrombocytopenia in patients with gastric cancer with a decrease in platelet count to <150,000/μL following gastrectomy and chemotherapy. Bone marrow aspiration/biopsy was performed with a single-use bone marrow aspiration needle (Allegiance Healthcare Corporation, Naperville, IL, USA) under local anesthesia. A single bone marrow aspirate was obtained from the posterior iliac crest before tumor manipulation. Ten milliliters of bone marrow were aspirated into a syringe containing 2500 IU of heparin and added to 10 mL of phosphate-buffered saline to prepare the cytospins. Hematoxylin-eosin, Giemsa, and reticulin staining were routinely performed on each slide in the pathology department. To verify the presence of tumor cells, immunohistochemical staining was performed when it was difficult to differentiate atypical cells from reactive cells.

### 2.3. Assessment of APCT Images

The APCTs of these patients were reviewed by two fellowship-trained board-certified radiologists with 6–18 years of experience. On APCT, if at least one suspicious osteolytic or sclerotic lesion was identified when two or three planes were reviewed, it was marked as suspicious; otherwise, it was considered negative. In the assessment of APCTs, two radiologists independently assessed the skeletal system. In cases where they provided discordant evaluations, lesions were reviewed through additional consensus processes.

Resulting from this process, 28 patients with both suspicious features on CT and biopsy-confirmed metastases, 12 with no suspicious features on CT but biopsy-confirmed metastasis, 13 with suspicious features on CT but normal bone marrow on biopsy, and 43 with no suspicious features on CT and normal bone marrow on biopsy were identified.

### 2.4. Region of Interest (ROI) Segmentation, Preprocessing, and Radiomic Feature Extraction

**Figure 2:**
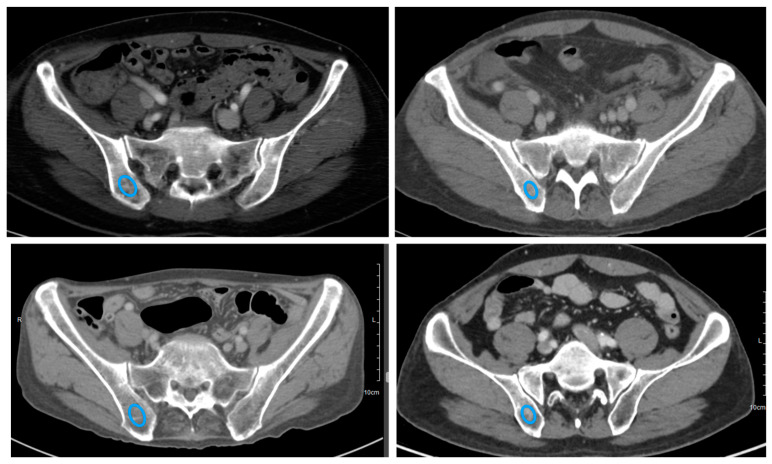
CT segmentation examples on contrast-enhanced axial scan for a patient with gastric cancer. Regions of interest were drawn in the right iliac bone on axial CT images (blue lines). Abbreviation: CT, computed tomography.

ROI segmentation was performed in a manual drawing with an area of 100 mm^2^ within the right iliac bone on contrast-enhanced axial APCT scans, corresponding to the same location where the bone marrow aspiration/biopsy was performed (Figure 2).

All images were preprocessed with normalization and resampling to prepare the images for radiomic analysis: (1) For the segmented ROIs, attenuation normalization was performed in a nonlinear manner into standardized intensity ranges for all subjects. (2) CT images were resampled with 1 mm pixel resampling.

The image dataset was read and transferred into MATLAB (MathWorks, Natick, MA, USA) format using in-house codes. For radiomic feature extraction, radiomic features were extracted from all of the CT images using MATLAB radiomics (https://github.com/mvallieres/radiomics accessed on 11 June 2024) [30]. All available first order features, shape features, gray-level co-occurrence matrix (GLCM) features, gray-level size zone matrix (GLSZM) features, gray-level run length matrix (GLRLM) features, neighborhood gray-tone difference matrix (NGTDM) features, and gray-level dependence matrix (GLDM) features were calculated.

### 2.5. Feature Categorization and Dimension Reduction

As mentioned previously, ROIs were drawn with a constant size in the posterior iliac spine, where bone marrow studies are typically performed. Therefore, diagnostic and shape features were excluded from the radiomic texture features. Accordingly, 43 radiomic texture features were extracted from each ROI. Additionally, the mean and standard deviation of the CT attenuation values (HU) within the ROI, which are conventionally obtained from CT scans, were selected as another set of variables. Finally, the overall CT features were divided into three feature sets: (1) mean and standard deviation values of attenuation in the ROI, (2) radiomic features extracted from the same ROI, and (3) combined features, which considered both CT features from the (1) mean and standard deviation, and (2) radiomic features.

The scikit-learn library in Python 3.11 (Python Software Foundation, Wilmington, DE, USA) was used for data handling, including the key feature selection process for radiomic features. To accurately assess the predictive power of each feature set while addressing potential issues from high-dimensional data, three different model types were developed based on the number of features selected using the Random Forest (RF) algorithm, including the top 10, 20, and 30 features. In addition, a fourth model type was developed that included all of the available features without any feature selection process. This approach resulted in 12 model types (three feature sets × four feature selection variations) that were compared to evaluate the impact of feature set composition on model performance. By systematically comparing models with varying numbers of selected features, we aimed to identify the most informative feature sets and the optimal number of features required for the accurate prediction of bone marrow metastasis in a clinical setting.

### 2.6. Development of Bone Marrow Metastasis Prediction Model

Based on the selected key features, five machine learning algorithms were developed: the K-Nearest Neighbor model, Decision Tree classifier, AdaBoost classifier, RF classifier, and Gradient-Boosting Machine. These algorithms were selected to represent a diverse range of machine learning techniques, each with its own strengths and weaknesses in capturing different aspects of the data. By employing multiple algorithms, we aimed to assess the overall performance of each feature set combination, rather than relying on the potential biases or limitations of a single algorithm.

The dataset was split into training and test sets, with 60% of the data used for training and the remaining 40% used for testing. This split was performed using stratified sampling based on the presence of bone metastasis in pathology and the presence of suspicious features on CT. During training, a stratified fivefold cross-validation approach was applied, maintaining the same stratification strategy. This process involved partitioning the training data into five equal-sized folds, with each fold serving as a validation set once while the model was trained on the remaining four folds. The optimal model and its hyperparameters were selected based on the area under the curve (AUC) of the receiver operating characteristic (ROC) curve, representing the true positive rate (sensitivity) plotted against the false positive rate (1—specificity) [31], which measured the model’s ability to predict bone marrow metastasis in patients with gastric cancer.

The performance of each trained model was evaluated on an independent test set using metrics such as the accuracy, precision, recall (sensitivity), F1-score, specificity, and AUC value, using a standard threshold of 0.5 for consistency and simplicity [32]. Accuracy indicates the overall correctness, precision (positive predictive value) is the ratio of true positives to total positive predictions, and recall is the ratio of true positives to actual positives. The F1-score, which is the harmonic mean of precision and recall, provides a balanced performance measure. Specificity is the ratio of true negatives to actual negatives, and AUC values provide discriminative power (range, 0.5–1.0). These metrics allowed for a comprehensive comparison of the predictive performance across different feature sets and model types, providing valuable insight into their potential for predicting bone marrow metastasis in patients with gastric cancer. The best-performing model in the test set was selected based on its AUC value.

### 2.7. Assessment of Bone Marrow Metastasis Prediction Model Performance with the External Validation Group

We validated the predictive performance of the best-performing model developed for the external validation group. Similarly, after calculating the accuracy, precision, recall (sensitivity), F1-score, specificity, and AUC, the predictive performance of the model was evaluated using the AUC of the ROC curve.

### 2.8. Statistical Analysis

Statistical analyses of clinical factors, including patient age at the time of gastric cancer diagnosis, platelet count, interval from diagnosis to bone marrow study, interval from CT scan to bone marrow study, and interval from platelet counting to bone marrow study, which are continuous variables, are expressed as means and standard deviations. Statistical significance was accepted when *p*-values were <0.05.

## 3. Results

### 3.1. Patient Characteristics

This study was conducted with contrast-enhanced APCT images taken prior to a bone marrow study in 96 patients (mean age, 58.4 ± 13.3 years; range, 28–85 years) with gastric cancer whose bone marrow pathology was confirmed through bone marrow aspiration/biopsy. Based on the pathologic results, which were the ground truth of the study, and APCT bone metastasis readings, which were assessed by two musculoskeletal radiologists, the patients were divided into four groups. Among the patients, 28 (29.2%) were pathology-positive (pathologically confirmed bone metastases) and CT-positive (presence of suspicious bone metastases on CT), 12 (12.5%) were pathology-positive and CT-negative (absence of suspicious bone metastases on CT), 13 (13.5%) were pathology-negative (absence of bone metastases confirmed by biopsy) and CT-positive, and 43 (44.8%) were pathology-negative and CT-negative. Table 1 presents the characteristics of the study population.

### 3.2. Diagnostic Performance of the Bone Marrow Metastasis Prediction Models

#### 3.2.1. In the Entire Patient Population

**Figure 3:**
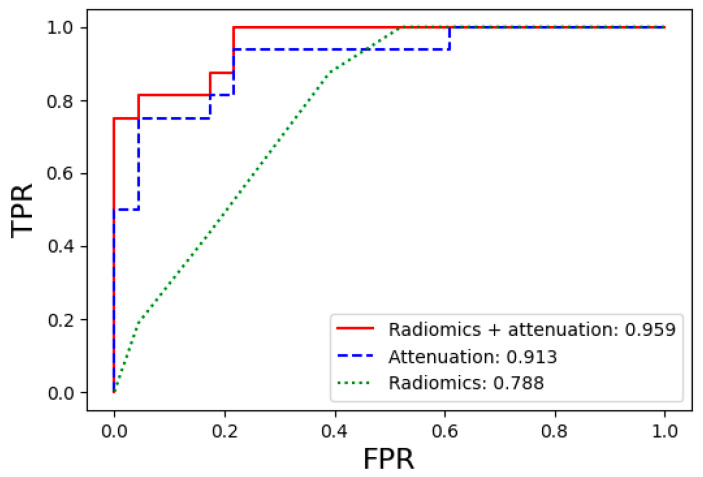
Comparison of the ROC curves for the prediction performance of bone marrow metastasis in the entire patient population using the optimal machine learning models. Abbreviations: ROC, receiver operating characteristic; TPR, true positive rate (sensitivity); FPR, false positive rate (1—specificity).

**Figure 4:**
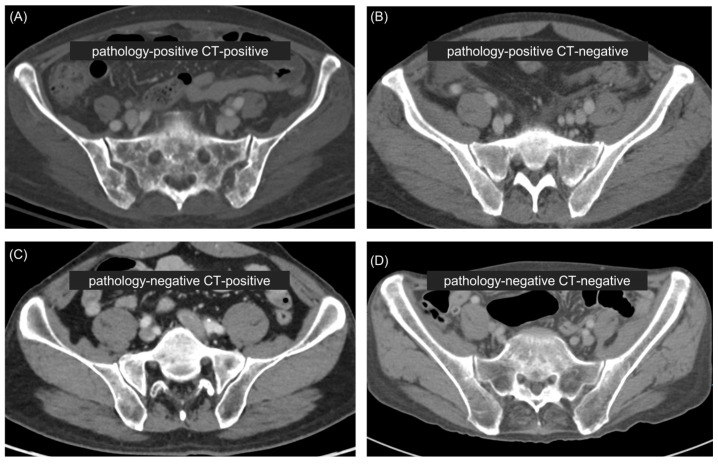
Representative cases. (**A**) CT image of a 67-year-old female patient shows heterogeneous densities in the sacrum and iliac bone, strongly suggesting bone metastasis. The bone marrow metastasis prediction model predicted bone metastasis based on the radiomic features. Pathological examination of the bone marrow confirmed bone metastasis. (**B**) CT image of a 55-year-old male patient shows no definite gross marrow changes; however, bone metastasis was pathologically confirmed. The prediction model predicted bone metastasis based on the radiomic features. (**C**) CT image of a 57-year-old male patient shows suspicious marrow inhomogeneity, but pathological examination confirmed no bone metastasis. The prediction model predicted no metastasis based on the radiomic features. (**D**) CT image of a 68-year-old male patient shows no marrow changes, and pathological examination confirmed no bone metastasis. The prediction model predicted no metastasis based on the radiomic features.

Table S1 shows the diagnostic performance of bone marrow metastasis prediction for the five machine learning models in the entire patient population across the radiomics, attenuation, and radiomics + attenuation datasets with four key feature selection levels. The overall performance of the models showed a clear trend in the order: radiomics + attenuation > attenuation > radiomics datasets. The best-performing model for bone marrow metastasis prediction was the RF classifier applied to the radiomics (including all 43 features) + attenuation model, achieving the highest performance, with an AUC of 0.96. The performance of the optimal models using the attenuation model and the radiomics model alone were not comparable to that of the radiomics + attenuation model, with AUC values of 0.91 and 0.78, respectively. The AUC, accuracy, sensitivity, specificity, precision, and F1-score values for each optimal model are presented in Table 2, and the ROC curves and AUC values are presented in Figure 3. Representative cases with CT images are presented in Figure 4.

#### 3.2.2. In the Pathology-Positive CT-Negative Group

**Figure 5:**
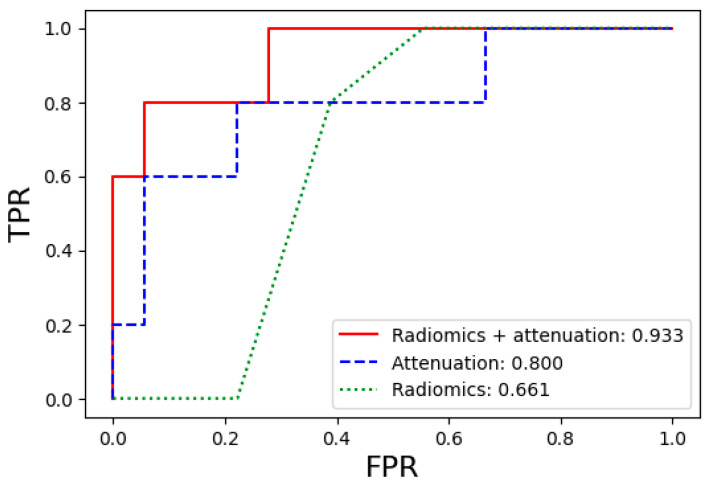
Comparison of the ROC curves for the prediction performance of bone marrow metastasis in the pathology-positive CT-negative group using the optimal machine learning models. Abbreviations: ROC, receiver operating characteristic; TPR, true positive rate (sensitivity); FPR, false positive rate (1—specificity).

In clinical practice, when patients with gastric cancer show equivocal signs of bone metastasis but recent CT scans do not reveal any suspicious features, the decision-making process to perform a bone marrow study becomes challenging, especially if the patient’s general condition has significantly deteriorated. In this light, we checked the performance of the bone marrow metastasis model, paying special attention to the pathology-positive CT-negative group, and the overall values are presented in Table S2. In the pathology-positive CT-negative group, the same trend appeared in the order of the radiomics + attenuation dataset, attenuation dataset, and radiomics dataset as the model performance in the entire patient group. For the optimal models, the performance differences between the radiomics + attenuation, attenuation, and radiomics datasets were more significant, with AUC values of 0.93, 0.80, and 0.66, respectively. The AUC, accuracy, sensitivity, specificity, precision, and F1-score values for each optimal model in the pathology-positive CT-negative group are presented in Table 3, and the ROC curves and AUC values are shown in Figure 5.

#### 3.2.3. In the External Validation Cohort

**Figure 6:**
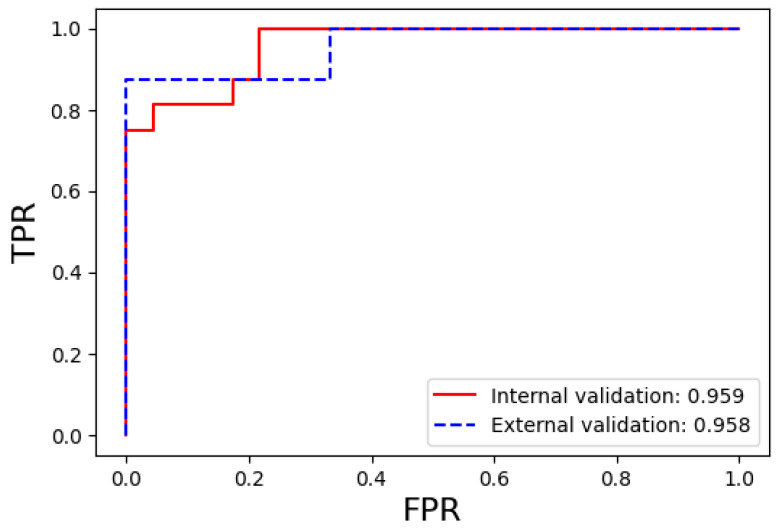
Comparison of the ROC curves for the prediction performance of bone marrow metastasis between the internal and external validation groups using the best-performing machine learning model. Abbreviations: ROC, receiver operating characteristic; TPR, true positive rate (sensitivity); FPR, false positive rate (1—specificity).

External validation was performed to ensure that the results obtained from the test set after training were applicable to other cohorts. As previously mentioned, we evaluated the performance of our proposed best-performing model in an additional group of 14 patients who met the inclusion criteria. The AUC, accuracy, sensitivity, specificity, precision, and F1-score values are presented in Table 4, along with a comparison with the internal validation cohort. The ROC curves and AUC values are shown in Figure 6. Here, we were able to confirm the excellent performance of our proposed best-performing model, with an AUC value of 0.96, which was consistent with the results from the internal validation cohort.

## 4. Discussion

Gastric cancer is a common cancer, predominantly in Asia. In Korea and Japan, it ranks fourth in overall cancer incidence and mortality rates. As mentioned previously, bone metastasis is one of the most common metastatic sites in patients with gastric cancer and can appear as metastatic lesions years after cancer treatment, making it a challenging issue in terms of treatment and prognosis. Therefore, several studies have been conducted to detect bone metastases in patients with gastric cancer during follow-up screening periods before symptoms such as severe pain caused by multiple bone metastases become evident. One such method in clinical practice is to perform a blind bone marrow study on the iliac bone when thrombocytopenia is observed during complete blood count follow-up. However, despite the rationale that cancer cell infiltration into the bone marrow can disrupt the marrow structure and cause hematological changes, the true predictive power of invasive bone marrow studies has been relatively low. In the approximately 17 years of patient data included in this study, it was found that out of 96 patients, 41 (42.7%) had bone metastases at the time of the bone marrow study. This indicated that the probability of detecting bone metastasis was less than half. Given this clinical background, this study focused on bone marrow metastasis in patients with gastric cancer.

For patients with gastric cancer, routine follow-up often involves APCT as a standard imaging modality. CT is a cost-effective, readily available, and time-efficient imaging modality. In addition to evaluating the operative site and lymph nodes, it can also be used to assess bone metastasis. However, its sensitivity and specificity for detecting bone metastases are not as high as in identifying marrow lesions. Therefore, alternative imaging modalities, such as PET-CT or MRI, are required. Nevertheless, the implementation of CT-based radiomics demonstrates overall high diagnostic accuracy, revealing image information that may not be discernible through visual readings by radiologists. Therefore, we aimed to effectively diagnose bone metastases in patients with gastric cancer using APCT. Specifically, we sought to devise a method for detecting invisible micrometastases, not only when suspicious osteolytic or sclerotic bone lesions are visible but also when they are not. Accordingly, this study was conducted by leveraging the potential of CT radiomics to analyze invisible texture features in imaging examinations.

Radiomic analysis can reflect tumor pathophysiology based on image-extracted information [33,34,35]. In musculoskeletal radiology, radiomics has been applied for differentiating benign and malignant tumors [36,37,38], tumor grade prediction [33,39,40,41], survival analysis [42], and treatment response [43]. In this study, we applied CT radiomic features, conventional CT attenuation (HU), and datasets combining various machine learning models. We identified an optimal model for diagnosing bone metastases using APCT. A model with high diagnostic performance for bone metastasis in the entire study population, achieving an AUC value of 0.96, was developed using only APCT without any other clinical information. This model utilized a dataset that combined conventional attenuation values and radiomic features. Additionally, we developed a sophisticated machine learning model with high diagnostic performance, achieving an AUC value of 0.93, by separately evaluating a group of patients with lesions that were invisible on CT but confirmed as metastases through bone marrow pathology, which is nearly impossible to detect with the human eye. Our proof-of-concept study shows promising results for radiomics applied to CT images for differentiating between bone metastases and metastasis-free bone in patients with gastric cancer. Importantly, radiomics enables this differentiation in a quantitative manner using CT images, showing only discrete abnormalities. Future advances include fully automatic bone segmentation frameworks for all patients with newly diagnosed gastric cancer, followed by the use of a radiomics classifier, allowing for an opportunistic screening-like approach for the early detection of bone metastases.

Bone marrow metastasis has a profound effect on the prognosis and treatment of patients with advanced-stage cancer [44]. Radiological examinations using CT, MRI, and PET-CT are the most commonly used noninvasive methods for diagnosing bone marrow metastases in cancer; however, bone metastases from malignancy may still be missed [45]. One of the main purposes of our study was to address the diagnostic and clinical dilemma of frequently missing metastatic bone disease on CT, which can only be detected with additional information from PET-CT imaging owing to the lack of morphological changes. It can be assumed that the human eye (without metabolic information) is not able to detect these relatively small lesions with no or subtle morphologic changes. In addition to the early detection of metastatic bone disease, timely and accurate prediction of bone metastases and identification of patients at high risk of bone metastases are highly desirable and could allow for the selection of patients most likely to benefit from targeted therapy. Recently, Wang et al. [32] developed and validated an MRI-based radiomics model for the individualized pretreatment prediction of bone metastases in patients with prostate cancer. We believe that the results of our study could add incremental value to diagnostic and treatment strategies, especially in patients with a high probability of bone metastasis, according to the aforementioned MRI-based radiomics nomogram [25] and microvessel density correlation [46]. Our study is a pioneering one that devised a method to predict bone metastasis on follow-up CT scans without performing additional invasive bone marrow studies or time-consuming and costly tests such as MRI, WBBS, or PET-CT/MRI to confirm further pathophysiology.

Our study has some limitations. First, the retrospective study design has inherent drawbacks, as only patients having gastric cancer with pathologically confirmed bone marrow metastasis were included. Second, it was not possible to identify an external validation set with patients possessing similar characteristics; therefore, temporal external validation was conducted instead. Third, although this study was conducted at a single institution and included a relatively small number of patients, it is significant considering that it focused on patients with gastric cancer over a period of approximately 20 years who had no prior diagnosis of bone metastasis and had undergone bone marrow studies for thrombocytopenia, with pathology providing the ground truth. In the future, we look forward to further multicenter studies with larger cohorts of patients to explore the applicability and potential expansion of this research.

## 5. Conclusions

Radiomic features derived from CT images serve as effective imaging biomarkers for predicting bone marrow metastasis, including microscopic bone metastasis, in patients with gastric cancer. These findings indicate the potential for their clinical utility in diagnosing and predicting bone marrow metastasis through routine evaluation of APCT images during follow-up examinations.

## Figures and Tables

**Table 1:** The characteristics of the study population.

		Pathology Bone Mets +		No Pathology Bone Mets −	
	All Patients	CT (+)	CT (−)	CT (+)	CT (−)
Variables	(*n* = 96)	(*n* = 28, 29.2%)	(*n* = 12, 12.5%)	(*n* = 13, 13.5%)	(*n* = 43, 44.8%)
Age, y	58.4 ± 13.4	51.4 ± 12.9	57.4 ± 9.7	65.6 ± 11.6	61.3 ± 13.5
Gender M:F	53:43	11:17	10:2	9:4	23:20
PLT, k	48.1 ± 37.0	32.5 ± 20.9	27.4 ± 19.6	63.3 ± 46.1	60.4 ± 40.4
Patho-Dx, d	1060.0 ± 1351.2	1099.7 ± 1778.7	889.0 ± 973.2	904.2 ± 1284.2	1129.3 ± 1170.9
Patho-CT, d	34.6 ± 70.4	8.7 ± 9.0	46.5 ± 60.1	87.8 ± 161.6	33.0 ± 44.7
Patho-PLT, d	22.3 ± 99.1	2.3 ± 2.4	3.8 ± 6.7	9.6 ± 25.5	44.4 ± 145.3

Values are mean ± SD or *n* (%). Abbreviations: Mets, metastasis; CT, computed tomography; PLT, platelet; Patho-Dx, the interval from gastric cancer diagnosis to bone marrow study; Patho-CT, the interval from CT scan to bone marrow study; Patho-PLT, the interval from platelet counting to bone marrow study.

**Table 2:** Diagnostic performance of optimal bone marrow metastasis prediction models in the entire patient population.

Dataset Type	Model	AUC	Accuracy	Sensitivity	Specificity	Precision	F1-Score
Radiomics + attenuation	RandomForest	0.959	0.846	0.813	0.870	0.813	0.846
Attenuation	KNeighbors	0.913	0.821	0.813	0.826	0.765	0.821
Radiomics	KNeighbors	0.788	0.667	0.438	0.826	0.636	0.652

Abbreviation: AUC, area under the curve.

**Table 3:** Diagnostic performance of optimal bone marrow metastasis prediction models in the pathology-positive CT-negative group.

Dataset Type	Model	AUC	Accuracy	Sensitivity	Specificity	Precision	F1-Score
Radiomics + Attenuation	RandomForest	0.933	0.826	0.800	0.833	0.571	0.835
Attenuation	KNeighbors	0.800	0.783	0.800	0.778	0.500	0.798
Radiomics	KNeighbors	0.661	0.609	0.000	0.778	0.000	0.592

Abbreviation: AUC, area under the curve.

**Table 4:** Diagnostic performance of the best-performing bone marrow metastasis prediction model in the external validation cohort compared to the internal validation cohort.

Dataset Type	Model	AUC	Accuracy	Sensitivity	Specificity	Precision
Internal validation	0.959	0.846	0.813	0.870	0.813	0.846
External validation	0.958	0.857	0.875	0.833	0.875	0.857

Abbreviation: AUC, area under the curve.

## Data Availability

The datasets presented in this article are not readily available because the data are part of an ongoing study.

## Supplementary Materials

The following supporting information can be downloaded at: https://www.mdpi.com/article/10.3390/diagnostics14151689/s1, Table S1: Diagnostic performance of the bone marrow metastasis prediction models in the entire patient population; Table S2: Diagnostic performance of the bone marrow metastasis prediction models in the pathology-positive CT-negative cohort.
