# Supplementary material for: Prediction of Bone Marrow Metastases Using Computed Tomography (CT) Radiomics in Patients with Gastric Cancer: Uncovering Invisible Metastases
Source: Diagnostics (Basel). 2024 Aug 5;14(15):1689. doi: 10.3390/diagnostics14151689 (PMC11312158; doi:10.3390/diagnostics14151689)
Supplement: Supplementary file 1 [file diagnostics-14-01689-s001.zip › diagnostics-3096295-supplementary.pdf]

Table S1. Diagnostic performance of the bone marrow metastasis prediction models in the **entire patient population**

Dataset Type	Model	AUC	Accuracy	Sensitivity	Specificity	Precision	F1-score
Radiomics + Attenuation	RandomForest	0.959	0.846	0.813	0.870	0.813	0.846
Radiomics + Attenuation (f10)	KNeighbors	0.954	0.897	0.750	1.000	1.000	0.894
Radiomics + Attenuation (f10)	RandomForest	0.951	0.897	0.938	0.870	0.833	0.898
Radiomics + Attenuation (f20)	KNeighbors	0.948	0.821	0.563	1.000	1.000	0.807
Radiomics + Attenuation (f20)	RandomForest	0.948	0.821	0.875	0.783	0.737	0.822
Radiomics + Attenuation (f30)	RandomForest	0.920	0.590	0.000	1.000	0.000	0.438
Radiomics + Attenuation (f10)	AdaBoost	0.920	0.821	0.813	0.826	0.765	0.821
Radiomics + Attenuation (f20)	AdaBoost	0.917	0.795	0.813	0.783	0.722	0.796
Radiomics + Attenuation (f20)	DecisionTree	0.916	0.872	0.750	0.957	0.923	0.869
Radiomics + Attenuation (f30)	KNeighbors	0.913	0.846	0.750	0.913	0.857	0.844
Attenuation	KNeighbors	0.913	0.821	0.813	0.826	0.765	0.821
Attenuation	RandomForest	0.908	0.846	0.875	0.826	0.778	0.847
Radiomics + Attenuation	AdaBoost	0.908	0.846	0.813	0.870	0.813	0.846
Radiomics + Attenuation (f30)	AdaBoost	0.908	0.846	0.813	0.870	0.813	0.846

Radiomics + Attenuation	GradientBoosting	0.905	0.846	0.938	0.783	0.750	0.847
Radiomics + Attenuation (f30)	GradientBoosting	0.902	0.769	0.813	0.739	0.684	0.771
Attenuation	DecisionTree	0.899	0.846	0.875	0.826	0.778	0.847
Radiomics + Attenuation	KNeighbors	0.894	0.872	0.813	0.913	0.867	0.871
Radiomics + Attenuation (f20)	GradientBoosting	0.891	0.795	0.875	0.739	0.700	0.796
Radiomics + Attenuation (f10)	GradientBoosting	0.880	0.692	0.688	0.696	0.611	0.694
Radiomics + Attenuation (f10)	DecisionTree	0.861	0.846	0.625	1.000	1.000	0.837
Attenuation	GradientBoosting	0.837	0.795	0.813	0.783	0.722	0.796
Attenuation	AdaBoost	0.811	0.769	0.750	0.783	0.706	0.770
Radiomics (f30)	KNeighbors	0.788	0.667	0.438	0.826	0.636	0.652
Radiomics	RandomForest	0.785	0.692	0.813	0.609	0.591	0.694
Radiomics (f30)	AdaBoost	0.783	0.744	0.625	0.826	0.714	0.740
Radiomics (f30)	RandomForest	0.779	0.590	0.000	1.000	0.000	0.438
Radiomics	AdaBoost	0.777	0.744	0.625	0.826	0.714	0.740
Radiomics + Attenuation (f30)	DecisionTree	0.776	0.769	0.813	0.739	0.684	0.771
Radiomics (f10)	RandomForest	0.774	0.692	0.750	0.652	0.600	0.695
Radiomics (f10)	AdaBoost	0.755	0.718	0.625	0.783	0.667	0.716
Radiomics + Attenuation	DecisionTree	0.753	0.769	0.688	0.826	0.733	0.768

Radiomics	KNeighbors	0.734	0.718	0.813	0.652	0.619	0.720
Radiomics	GradientBoosting	0.713	0.692	0.750	0.652	0.600	0.695
Radiomics (f30)	GradientBoosting	0.712	0.718	0.750	0.696	0.632	0.720
Radiomics (f20)	RandomForest	0.705	0.590	0.000	1.000	0.000	0.438
Radiomics (f20)	GradientBoosting	0.705	0.718	0.750	0.696	0.632	0.720
Radiomics (f20)	DecisionTree	0.701	0.692	0.688	0.696	0.611	0.694
Radiomics (f10)	GradientBoosting	0.696	0.718	0.750	0.696	0.632	0.720
Radiomics	DecisionTree	0.694	0.692	0.688	0.696	0.611	0.694
Radiomics (f10)	DecisionTree	0.694	0.692	0.688	0.696	0.611	0.694
Radiomics (f30)	DecisionTree	0.694	0.692	0.688	0.696	0.611	0.694
Radiomics (f10)	KNeighbors	0.685	0.590	0.438	0.696	0.500	0.585
Radiomics (f20)	AdaBoost	0.667	0.641	0.813	0.522	0.542	0.639
Radiomics (f20)	KNeighbors	0.666	0.615	0.563	0.652	0.529	0.617
Radiomics + Attenuation	RandomForest	0.959	0.846	0.813	0.870	0.813	0.846
Radiomics + Attenuation (f10)	KNeighbors	0.954	0.897	0.750	1.000	1.000	0.894
Radiomics + Attenuation (f10)	RandomForest	0.951	0.897	0.938	0.870	0.833	0.898
Radiomics + Attenuation (f20)	KNeighbors	0.948	0.821	0.563	1.000	1.000	0.807
Radiomics + Attenuation (f20)	RandomForest	0.948	0.821	0.875	0.783	0.737	0.822

Radiomics + Attenuation (f30)	RandomForest	0.920	0.590	0.000	1.000	0.000	0.438
Radiomics + Attenuation (f10)	AdaBoost	0.920	0.821	0.813	0.826	0.765	0.821
Radiomics + Attenuation (f20)	AdaBoost	0.917	0.795	0.813	0.783	0.722	0.796
Radiomics + Attenuation (f20)	DecisionTree	0.916	0.872	0.750	0.957	0.923	0.869
Radiomics + Attenuation (f30)	KNeighbors	0.913	0.846	0.750	0.913	0.857	0.844
Attenuation	KNeighbors	0.913	0.821	0.813	0.826	0.765	0.821
Attenuation	RandomForest	0.908	0.846	0.875	0.826	0.778	0.847
Radiomics + Attenuation	AdaBoost	0.908	0.846	0.813	0.870	0.813	0.846
Radiomics + Attenuation (f30)	AdaBoost	0.908	0.846	0.813	0.870	0.813	0.846
Radiomics + Attenuation	GradientBoosting	0.905	0.846	0.938	0.783	0.750	0.847
Radiomics + Attenuation (f30)	GradientBoosting	0.902	0.769	0.813	0.739	0.684	0.771
Attenuation	DecisionTree	0.899	0.846	0.875	0.826	0.778	0.847
Radiomics + Attenuation	KNeighbors	0.894	0.872	0.813	0.913	0.867	0.871
Radiomics + Attenuation (f20)	GradientBoosting	0.891	0.795	0.875	0.739	0.700	0.796
Radiomics + Attenuation (f10)	GradientBoosting	0.880	0.692	0.688	0.696	0.611	0.694
Radiomics + Attenuation (f10)	DecisionTree	0.861	0.846	0.625	1.000	1.000	0.837
Attenuation	GradientBoosting	0.837	0.795	0.813	0.783	0.722	0.796
Attenuation	AdaBoost	0.811	0.769	0.750	0.783	0.706	0.770

Radiomics (f30)	KNeighbors	0.788	0.667	0.438	0.826	0.636	0.652
Radiomics	RandomForest	0.785	0.692	0.813	0.609	0.591	0.694
Radiomics (f30)	AdaBoost	0.783	0.744	0.625	0.826	0.714	0.740
Radiomics (f30)	RandomForest	0.779	0.590	0.000	1.000	0.000	0.438
Radiomics	AdaBoost	0.777	0.744	0.625	0.826	0.714	0.740
Radiomics + Attenuation (f30)	DecisionTree	0.776	0.769	0.813	0.739	0.684	0.771
Radiomics (f10)	RandomForest	0.774	0.692	0.750	0.652	0.600	0.695
Radiomics (f10)	AdaBoost	0.755	0.718	0.625	0.783	0.667	0.716
Radiomics + Attenuation	DecisionTree	0.753	0.769	0.688	0.826	0.733	0.768
Radiomics	KNeighbors	0.734	0.718	0.813	0.652	0.619	0.720
Radiomics	GradientBoosting	0.713	0.692	0.750	0.652	0.600	0.695
Radiomics (f30)	GradientBoosting	0.712	0.718	0.750	0.696	0.632	0.720
Radiomics (f20)	RandomForest	0.705	0.590	0.000	1.000	0.000	0.438
Radiomics (f20)	GradientBoosting	0.705	0.718	0.750	0.696	0.632	0.720
Radiomics (f20)	DecisionTree	0.701	0.692	0.688	0.696	0.611	0.694
Radiomics (f10)	GradientBoosting	0.696	0.718	0.750	0.696	0.632	0.720
Radiomics	DecisionTree	0.694	0.692	0.688	0.696	0.611	0.694
Radiomics (f10)	DecisionTree	0.694	0.692	0.688	0.696	0.611	0.694

Radiomics (f30)	DecisionTree	0.694	0.692	0.688	0.696	0.611	0.694
Radiomics (f10)	KNeighbors	0.685	0.590	0.438	0.696	0.500	0.585
Radiomics (f20)	AdaBoost	0.667	0.641	0.813	0.522	0.542	0.639
Radiomics (f20)	KNeighbors	0.666	0.615	0.563	0.652	0.529	0.617

Abbreviation: AUC, area under the curve; (f), key feature selection number.

Table S2. Diagnostic performance of the bone marrow metastasis prediction models in the pathology-positive CT-negative cohort

Dataset Type	Model	AUC	Accuracy	Sensitivity	Specificity	Precision	F1-score
Radiomics + Attenuation	RandomForest	0.933	0.826	0.800	0.833	0.571	0.835
Radiomics + Attenuation (f10)	KNeighbors	0.900	0.913	0.600	1.000	1.000	0.904
Radiomics + Attenuation (f10)	RandomForest	0.867	0.826	0.800	0.833	0.571	0.835
Radiomics + Attenuation (f20)	KNeighbors	0.911	0.826	0.200	1.000	1.000	0.777
Radiomics + Attenuation (f20)	RandomForest	0.878	0.739	0.800	0.722	0.444	0.760
Radiomics + Attenuation (f30)	RandomForest	0.850	0.783	0.000	1.000	0.000	0.687
Radiomics + Attenuation (f10)	AdaBoost	0.817	0.783	0.800	0.778	0.500	0.798
Radiomics + Attenuation (f20)	AdaBoost	0.800	0.696	0.600	0.722	0.375	0.717
Radiomics + Attenuation (f20)	DecisionTree	0.811	0.870	0.600	0.944	0.750	0.864
Radiomics + Attenuation (f30)	KNeighbors	0.889	0.826	0.600	0.889	0.600	0.826
Attenuation	KNeighbors	0.800	0.783	0.800	0.778	0.500	0.798
Attenuation	RandomForest	0.800	0.783	0.800	0.778	0.500	0.798
Radiomics + Attenuation	AdaBoost	0.783	0.783	0.600	0.833	0.500	0.789
Radiomics + Attenuation (f30)	AdaBoost	0.783	0.783	0.600	0.833	0.500	0.789
Radiomics + Attenuation	GradientBoosting	0.822	0.739	0.800	0.722	0.444	0.760

Radiomics + Attenuation (f30)	GradientBoosting	0.822	0.696	0.800	0.667	0.400	0.722
Attenuation	DecisionTree	0.844	0.783	0.800	0.778	0.500	0.798
Radiomics + Attenuation	KNeighbors	0.833	0.870	0.800	0.889	0.667	0.874
Radiomics + Attenuation (f20)	GradientBoosting	0.789	0.696	0.800	0.667	0.400	0.722
Radiomics + Attenuation (f10)	GradientBoosting	0.800	0.609	0.600	0.611	0.300	0.642
Radiomics + Attenuation (f10)	DecisionTree	0.844	0.913	0.600	1.000	1.000	0.904
Attenuation	GradientBoosting	0.700	0.739	0.800	0.722	0.444	0.760
Attenuation	AdaBoost	0.728	0.739	0.800	0.722	0.444	0.760
Radiomics (f30)	KNeighbors	0.661	0.609	0.000	0.778	0.000	0.592
Radiomics	RandomForest	0.678	0.696	1.000	0.611	0.417	0.722
Radiomics (f30)	AdaBoost	0.678	0.696	0.400	0.778	0.333	0.705
Radiomics (f30)	RandomForest	0.639	0.783	0.000	1.000	0.000	0.687
Radiomics	AdaBoost	0.678	0.696	0.400	0.778	0.333	0.705
Radiomics + Attenuation (f30)	DecisionTree	0.633	0.652	0.600	0.667	0.333	0.680
Radiomics (f10)	RandomForest	0.656	0.565	0.600	0.556	0.273	0.603
Radiomics (f10)	AdaBoost	0.667	0.696	0.600	0.722	0.375	0.717
Radiomics + Attenuation	DecisionTree	0.633	0.696	0.400	0.778	0.333	0.705
Radiomics	KNeighbors	0.689	0.652	0.600	0.667	0.333	0.680

Radiomics	GradientBoosting	0.567	0.565	0.600	0.556	0.273	0.603
Radiomics (f30)	GradientBoosting	0.567	0.609	0.600	0.611	0.300	0.642
Radiomics (f20)	RandomForest	0.622	0.783	0.000	1.000	0.000	0.687
Radiomics (f20)	GradientBoosting	0.544	0.609	0.600	0.611	0.300	0.642
Radiomics (f20)	DecisionTree	0.544	0.565	0.400	0.611	0.222	0.600
Radiomics (f10)	GradientBoosting	0.567	0.609	0.600	0.611	0.300	0.642
Radiomics	DecisionTree	0.544	0.565	0.400	0.611	0.222	0.600
Radiomics (f10)	DecisionTree	0.544	0.565	0.400	0.611	0.222	0.600
Radiomics (f30)	DecisionTree	0.544	0.565	0.400	0.611	0.222	0.600
Radiomics (f10)	KNeighbors	0.517	0.565	0.200	0.667	0.143	0.589
Radiomics (f20)	AdaBoost	0.550	0.522	0.600	0.500	0.250	0.562
Radiomics (f20)	KNeighbors	0.578	0.609	0.400	0.667	0.250	0.636
Radiomics + Attenuation	RandomForest	0.933	0.826	0.800	0.833	0.571	0.835
Radiomics + Attenuation (f10)	KNeighbors	0.900	0.913	0.600	1.000	1.000	0.904
Radiomics + Attenuation (f10)	RandomForest	0.867	0.826	0.800	0.833	0.571	0.835
Radiomics + Attenuation (f20)	KNeighbors	0.911	0.826	0.200	1.000	1.000	0.777
Radiomics + Attenuation (f20)	RandomForest	0.878	0.739	0.800	0.722	0.444	0.760
Radiomics + Attenuation (f30)	RandomForest	0.850	0.783	0.000	1.000	0.000	0.687

Radiomics + Attenuation (f10)	AdaBoost	0.817	0.783	0.800	0.778	0.500	0.798
Radiomics + Attenuation (f20)	AdaBoost	0.800	0.696	0.600	0.722	0.375	0.717
Radiomics + Attenuation (f20)	DecisionTree	0.811	0.870	0.600	0.944	0.750	0.864
Radiomics + Attenuation (f30)	KNeighbors	0.889	0.826	0.600	0.889	0.600	0.826
Attenuation	KNeighbors	0.800	0.783	0.800	0.778	0.500	0.798
Attenuation	RandomForest	0.800	0.783	0.800	0.778	0.500	0.798
Radiomics + Attenuation	AdaBoost	0.783	0.783	0.600	0.833	0.500	0.789
Radiomics + Attenuation (f30)	AdaBoost	0.783	0.783	0.600	0.833	0.500	0.789
Radiomics + Attenuation	GradientBoosting	0.822	0.739	0.800	0.722	0.444	0.760
Radiomics + Attenuation (f30)	GradientBoosting	0.822	0.696	0.800	0.667	0.400	0.722
Attenuation	DecisionTree	0.844	0.783	0.800	0.778	0.500	0.798
Radiomics + Attenuation	KNeighbors	0.833	0.870	0.800	0.889	0.667	0.874
Radiomics + Attenuation (f20)	GradientBoosting	0.789	0.696	0.800	0.667	0.400	0.722
Radiomics + Attenuation (f10)	GradientBoosting	0.800	0.609	0.600	0.611	0.300	0.642
Radiomics + Attenuation (f10)	DecisionTree	0.844	0.913	0.600	1.000	1.000	0.904
Attenuation	GradientBoosting	0.700	0.739	0.800	0.722	0.444	0.760
Attenuation	AdaBoost	0.728	0.739	0.800	0.722	0.444	0.760
Radiomics (f30)	KNeighbors	0.661	0.609	0.000	0.778	0.000	0.592

Radiomics	RandomForest	0.678	0.696	1.000	0.611	0.417	0.722
Radiomics (f30)	AdaBoost	0.678	0.696	0.400	0.778	0.333	0.705
Radiomics (f30)	RandomForest	0.639	0.783	0.000	1.000	0.000	0.687
Radiomics	AdaBoost	0.678	0.696	0.400	0.778	0.333	0.705
Radiomics + Attenuation (f30)	DecisionTree	0.633	0.652	0.600	0.667	0.333	0.680
Radiomics (f10)	RandomForest	0.656	0.565	0.600	0.556	0.273	0.603
Radiomics (f10)	AdaBoost	0.667	0.696	0.600	0.722	0.375	0.717
Radiomics + Attenuation	DecisionTree	0.633	0.696	0.400	0.778	0.333	0.705
Radiomics	KNeighbors	0.689	0.652	0.600	0.667	0.333	0.680
Radiomics	GradientBoosting	0.567	0.565	0.600	0.556	0.273	0.603
Radiomics (f30)	GradientBoosting	0.567	0.609	0.600	0.611	0.300	0.642
Radiomics (f20)	RandomForest	0.622	0.783	0.000	1.000	0.000	0.687
Radiomics (f20)	GradientBoosting	0.544	0.609	0.600	0.611	0.300	0.642
Radiomics (f20)	DecisionTree	0.544	0.565	0.400	0.611	0.222	0.600
Radiomics (f10)	GradientBoosting	0.567	0.609	0.600	0.611	0.300	0.642
Radiomics	DecisionTree	0.544	0.565	0.400	0.611	0.222	0.600
Radiomics (f10)	DecisionTree	0.544	0.565	0.400	0.611	0.222	0.600
Radiomics (f30)	DecisionTree	0.544	0.565	0.400	0.611	0.222	0.600

Radiomics (f10)	KNeighbors	0.517	0.565	0.200	0.667	0.143	0.589
Radiomics (f20)	AdaBoost	0.550	0.522	0.600	0.500	0.250	0.562
Radiomics (f20)	KNeighbors	0.578	0.609	0.400	0.667	0.250	0.636

Abbreviation: AUC, area under the curve; (f), key feature selection number.
